# Fabrication and Characterization of Superhydrophobic Al-Based Surface Used for Finned-Tube Heat Exchangers

**DOI:** 10.3390/ma15093060

**Published:** 2022-04-22

**Authors:** Ran Li, Zanshe Wang, Meijuan Chen, Zhang Li, Xiaowei Luo, Weizhen Lu, Zhaolin Gu

**Affiliations:** 1 School of Human Settlements and Civil Engineering, Xi’an Jiaotong University, Xi’an 710049, China; lillianliran@stu.xjtu.edu.cn (R.L.); wangzs@mail.xjtu.edu.cn (Z.W.); chenmeijuan@mail.xjtu.edu.cn (M.C.); lizhang@stu.xjtu.edu.cn (Z.L.); 2 Department of Architecture and Civil Engineering, City University of Hong Kong, Hong Kong 999077, China; xiaowluo@cityu.edu.hk; 3 Zhejiang Research Institute of Xi’an Jiaotong University, Hangzhou 311215, China

**Keywords:** superhydrophobic fins, contact angle, Al alloy, heat exchanger, durability

## Abstract

Enhancing the heat transfer performance of heat exchangers is one of the main methods to reduce energy consumption and carbon emissions in heating, ventilation, air-conditioning and refrigeration (HVAC&R) systems. Wettability modified surfaces developed gradually may help. This study aims to improve the performance of heat exchangers from the perspective of component materials. The facile and cost-effective fabrication method of superhydrophobic Al-based finned-tube heat exchangers with acid etching and stearic acid self-assembly was proposed and optimized in this study, so that the modified Al fins could achieve stronger wettability and durability. The effect of process parameters on the wettability of the Al fins was by response surface methodology (RSM) and variance analysis. Then, the modified fins were characterized by field-emission scanning electron microscopy (FE-SEM), 3D topography profiler, X-ray photoelectron spectroscopy (XPS), and Fourier transform infrared spectroscopy (FTIR), respectively. The durability of the superhydrophobic fins was investigated by air exposure, corrosion resistance, and mechanical robustness experiments. The RSM and variance analysis demonstrated that a water contact angle (WCA) of 166.9° can be obtained with the etching time in 2 mol/L HCl solution of 10.5 min, the self-assembly time in the stearic acid ethanol solution of 48 h, and drying under 73.0 °C. The surface morphology showed suitable micro-nano structures with a mean roughness (Ra) of 467.58 nm and a maximum peak-to-valley vertical distance (Rt) of 4.095 μm. The chemical component demonstrated the self-assembly of an alkyl chain. The WCAs declined slightly in durability experiments, which showed the feasibility of the superhydrophobic heat exchangers under actual conditions.

## 1. Introduction

In recent years, with the gradual improvement in people’s requirements for environmental thermal comfort, built environment control equipment such as air-conditioners and air source heat pumps (ASHP) have been widely used, leading to an increase in building energy consumption. According to the International Energy Agency (IEA), the buildings and the construction industries account for nearly 40% of the global CO_2_ emissions [1]. The Al-based finned-tube heat exchanger is a common component used in heating, ventilation, air-conditioning and refrigeration (HVAC&R) systems. Improving its heat transfer performance is significant to enhancing the energy efficiency of HVAC&R systems. Usually, reducing the heat transfer resistance and increasing the convective heat transfer coefficient is crucial to improving heat transfer performance [2]. In actual operation, many problems such as frost on the evaporator in winter conditions [3,4], fouling on the air-side surface [5,6], corrosion of the fins [7], etc., cause the heat transfer resistance to increase and the energy efficiency of the heat exchangers to decline.

Since Neinhuis and Barthlott [8] proposed the Lotus-effect based on the anti-pollution property of lotus leaf surface, studies on the hydrophobic/superhydrophobic surface imitating lotus-leaf characteristics have been developing vigorously. The superhydrophobic property of the surface is characterized by the water contact angle (WCA) and the contact angle hysteresis (CAH). When the contact angle is greater than 150° and the contact angle hysteresis is less than 10°, the surface can be defined as a superhydrophobic surface [9]. Superhydrophobic surfaces showed considerable potential in medical implants [10], fabric anti-fouling [11], water and oil separation [12] and so on. Notably, the properties of anti-icing [13,14,15,16], surface drag reduction [17,18,19], self-cleaning [20,21,22], and anti-corrosion [23,24,25] are well suited to solve the problems of heat exchangers mentioned before. Therefore, this study hopes to apply the advantages of superhydrophobic surfaces to finned-tube heat exchangers of HVAC&R systems, providing a breakthrough point for improving their energy efficiency.

The two-step methods have been relatively mature in the existing fabrication method [26,27,28,29,30,31,32] to prepare superhydrophobic Al surfaces. Usually, the first step is to produce micro/nano-structures on the substrate material, and the second is to make low surface free energy materials combine with the micro-nano structures [33]. In previous studies, the fabrication by stearic acid modification on Al surfaces with suitable micro-nano structures shows a cost-effective advantage. Wang et al. [34] studied the ice accretion phenomena on three kinds of prepared Al-based surfaces with different hydrophobic properties. Among them, the superhydrophobic Al surface fabricated by etching with a 30% HCl aqueous solution and immersed in an acetone stearic acid solution showed better ice phobic performance. The WCA of the superhydrophobic Al surface was larger than 150°. Zhu [27] fabricated superhydrophobic surfaces on Al substrate by creating the rough surface with machine cutting and stearic acid modification. The resulting surfaces had contact angles of more than 150°. Chen et al. [35] obtained a superhydrophobic Al surface which owned a WCA of 150° and a sliding angle of 8° by immersing the substrates in the aqueous solution of HCl and stearic acid. Peng et al. [36] also used chemical etching and stearic acid ethanol solution to prepare superhydrophobic Al surface. Overall, the surfaces modified with stearic acid can obtain WCAs above 150°. The acid etching process before stearic acid modification could obtain relatively larger contact angles [37] compared with other methods of fabricating microstructure on Al substrates.

However, although a number of studies explored superhydrophobic surfaces with corrosion resistance and mechanical robustness [23,28,38,39,40,41], they did not address specific industrial components, which means the utility of these fabrication methods for heat exchangers in HVAC&R systems is unknown. On the other hand, there are only a few studies focused on superhydrophobic heat exchangers, and the research focused on the evaluation of heat transfer performance [42,43,44]. Edalatpour et al. [45] pointed out that the application of superhydrophobic heat exchangers were not widespread due to a significant limitation that most of the superhydrophobic surfaces may lose efficiency over time. Therefore, a research gap between the fabrication process and durability of the practical application still remains in the usage of superhydrophobic heat exchangers.

In general, although relevant studies involving materials and surface treatment have discussed the mechanism of surface wettability, they did not combine the certain engineering components with practical application conditions to discuss the feasibility and durability. In this study, we first optimized the two-step method of acid etching and stearic acid modification for preparing superhydrophobic Al-based finned-tube heat exchangers, giving a transparent and optimized fabrication process and parameters. This fabrication method breaks through the limit of WCAs of most stearic acid modified Al surfaces, making the WCA stably reach more than 160°. Then, the durability of the modified Al fins was discussed to ensure the application feasibility of the proposed method used in HVAC&R systems.

## 2. Materials and Methods

### 2.1. Materials

The Al alloy substrates were the 1060 Al series with the size of 40 × 40 × 0.2 mm (purity of Al is 99.6%, provided by Shenzhen Hongwang Mould Co., Ltd., Shenzhen, China). Na_2_CO_3_ (AR grade) and Na_3_PO_4_ (AR grade) used in mixed alkali solution pre-treatment, and MnH_4_P_2_O_8_·2H_2_O (AR grade) used in acid solution pre-treatment were obtained from Shanghai Macklin Biochemical Co., Ltd., Shanghai, China. Ethanol (AR, 99.7%) was purchased from Tianli Chemical Reagent Co., Ltd., Tianjin, China. Aqueous HCl (AR grade) was obtained from Chengdu Chronch Chemical Co., Ltd., Chengdu, China. Stearic acid (AR grade) was purchased from Tianjin Dingsheng Chemical Industry Co., Ltd., Tianjin, China. Deionized water was obtained locally.

Among them, the mass concentration of Na_2_CO_3_ and Na_3_PO_4_ in the mixed alkaline solution are 10.0 g/L and 23.1 g/L, respectively. The mass concentration of MnH_4_P_2_O_8_·2H_2_O and the volume concentration of ethanol in the acid solution are 0.6 g/L and 50 mL/L, respectively. The aqueous HCl solutions were prepared at 1 mol/L, 2 mol/L and 3 mol/L, respectively. The concentration of stearic acid ethanol solution is 0.02 mol/L.

### 2.2. Surface Fabrication Process

**Figure 1:**
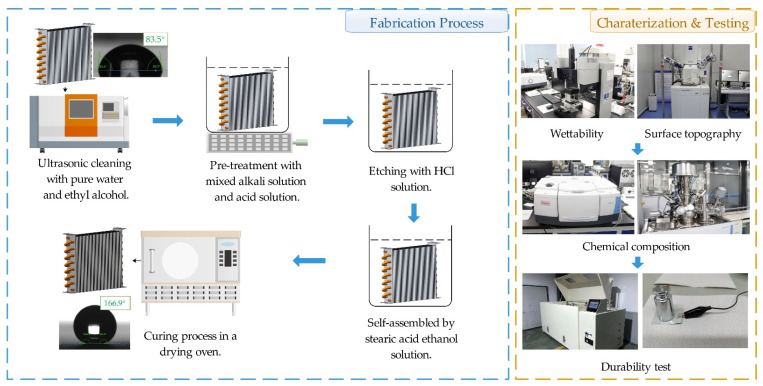
Research workflow of the superhydrophobic Al-based finned-tube heat exchanger.

The Al fins were firstly pretreated in deionized water and ethanol for 10 min, respectively, with an ultrasonic cleaning machine (JP-020, Jiemeng, Shenzhen, China) to remove the surface impurities. Then, the fins were immersed in mixed alkaline solution and acid solution for 3 min under 45 °C, respectively, to further remove foreign matter. Next, etched the fins by HCl solution to obtain a micron-scale surface structure, and then rendered superhydrophobic by the self-assembly of stearic acid under room temperature. Finally, the curing process was carried out in a drying oven (WGL-45B, TAISITE, Tianjin, China) under 70 °C to 90 °C. The fabrication process is shown in Figure 1. The single-factor experiment concentrated on the concentration of HCl aqueous solution and the response surface method (RSM) were employed to investigate the influence of multiple variables [46], and to get a credible process with optimized parameters to fabricate the superhydrophobic Al alloy surface. Design EXPERT V8 was used for the RSM experimental design.

### 2.3. Surface Characterization

The water contact angle (WCA) and the contact angle hysteresis (CAH) of Al fins were measured at room temperature using a drop shape analyzer system (KRUSS DSA 100E, KRÜSS GmbH, Hamburg, Germany) under the sessile drop model. When testing the WCA, 4 µL water droplets were released at six different spots over the test surface, and the average values were considered as the result. When testing the CAH, the needle of the instrument first pushed out a 4 µL water droplet and contacted the measuring surface. Then, the advancing contact angle and receding contact angle were obtained by increasing or decreasing the volume of the droplet. The difference between the advancing and receding contact angle was the CAH.

The surface micro-topography was observed using field-emission scanning electron microscopy (FE-SEM) ZEISS Gemini 500. The surface 3D topography and surface roughness were measured with 3D topography profiler Bruker Contour GT-X. The chemical composition was evaluated by Fourier Transform Infrared (FTIR) spectrometer (Nicolet iS50) and X-ray photoelectron spectroscopy (XPS, Thermo ESCALAB 250XI).

### 2.4. Investigation of Durability

The surface durability was investigated through air exposure, corrosion and mechanical robustness test. The air exposure test was carried out by exposing the superhydrophobic heat exchangers to the air for one year, and the WCAs of the fin sample were measured at intervals to obtain the decay trend of super-hydrophobicity. The corrosion resistance was evaluated by the neutral salt spray (NSS) test with a salt solution spray machine (YWX-120C, Marit, Wuxi, China) and by immersing the fins in acidic and alkaline solutions with different pH values. The accelerated corrosion experiment using NSS test machine was carried out with a saltwater solution with a concentration of 50 ± 5 g/L and a pH value of 6.5~7.2. The temperature in the test box was 35 ± 2 °C. The humidity in the test box was greater than 95%, and the fog reduction volume was 1~2 mL/(h·cm^2^). The Al samples were sprayed with the salt solution for 12 h, 24 h, 48 h and 72 h, then observed the surface corrosion state. The salt crystals were then washed away with deionized water, and their contact angles were measured. The pH stability test was conducted by immersing the Al fins in solutions with different pH values of 3, 5, 7, 9,11, respectively. The contact angles of each fin were measured after immersing for 6, 12, 20, and 30 h. The mechanical robustness of the surface was assessed by tangential wear with a sandpaper of 240-grit. A modified Al fin with an initial WCA of 163.6°were placed on the sandpaper and pulled at a uniform speed under a load of 200 g. The load was pulled for 10 cm and repeated ten times.

## 3. Results and Discussion

### 3.1. Fabrication Process Optimization

#### 3.1.1. Influence of Etching Solution Concentration

**Figure 2:**
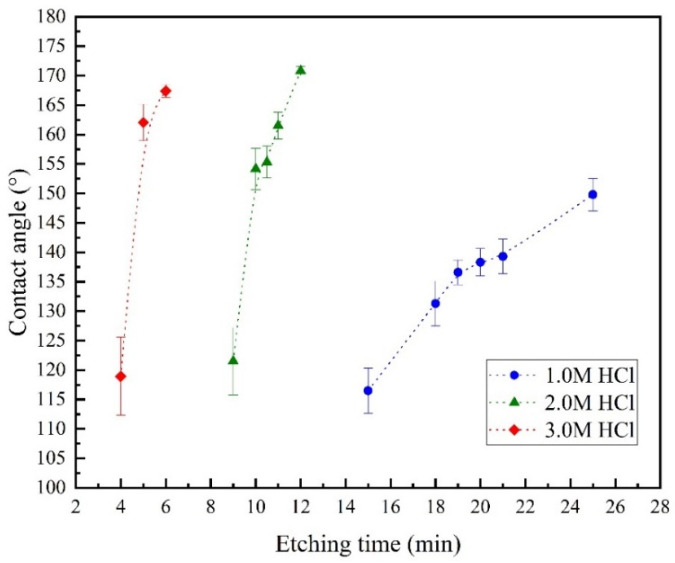
The relationship between the concentration of the etching solution and the etching time.

The concentration of aqueous HCl solution and the etching time are closely related to the etching degree of the Al sheet. If the etching degree is insufficient, a suitable micron-scale rough structure cannot be obtained. If the etching degree is too large, the mass of the Al sheet is seriously lost, causing a reduction in the mechanical strength and even the dissolution of the sample. In this experiment, we chose 1.0, 2.0, and 3.0 mol/L aqueous HCl solution. For 1.0 mol/L aqueous HCl solution, the etching times were 15, 18, 19, 20, 21, and 25 min. For 2.0 mol/L aqueous HCl solution, the etching times were 9, 10, 10.5, 11, and 12 min. For 3.0 mol/L aqueous HCl solution, the etching times were 4, 5, and 6 min. The maximum etching time was determined according to the extent of reaction of the Al sheet during the etching process. After the etching process, the samples were rinsed and dried. Then, they were immersed in a solution of 0.02 mol/L stearic acid and ethanol for deposition. After 24 h, the samples were thoroughly rinsed with deionized water and then dried in an oven at 80 °C for 30 min. The WCAs were measured after cooling the samples to room temperature. The results are shown in Figure 2.

The error bars mean the standard deviation, which can be considered as the surface uniformity after etching. For 1.0 mol/L aqueous HCl solution, the etching time was longer, and it took more than 25 min to achieve super-hydrophobicity (the static WCA was greater than 150°). When 2.0 and 3.0 mol/L aqueous HCl solutions were used for etching, the time required to achieve super-hydrophobicity was greatly reduced. When the etching time reached 12 and 6 min, respectively, the etching reaction became violent, and a lot of heat was released. At that time, the samples were taken out and weighed after rinsing and drying. The mass loss reached 40%, so it was regarded as the upper limit of the etching time. In actual operation, it was found that when 3.0 mol/L aqueous HCl solution was used for etching, the reaction was too rapid, so the time interval between the samples reaching super-hydrophobicity to over-dissolution was too short to control the etching time. Therefore, 2.0 mol/L was chosen as the etching solution concentration for the subsequent experiments. According to the standard deviation of the WCA, as the reaction time increases, the degree of surface etching tends to be uniform. At the same time, considering the quality loss of the Al alloy fins, the etching time is determined to be 10 min~11 min to obtain a superhydrophobic surface, and the standard deviation is 1~2%.

#### 3.1.2. Process Optimization Based on RSM

Based on the above single-factor experimental results, the three factors: etching time, deposition time, and drying temperature, were chosen for the response surface analysis to determine the process parameters, and the Box–Behnken design was selected as the design method. Each factor and its design value are listed in Table 1. The response value is the average value of the measured WCAs. The experimental plan and results are shown in Table 2.

The multiple linear regression fitting was performed on the experimental data in Table 2, and the multiple regression response surface model at the static WCA was obtained. Then, the regression model was established, as shown in Equation (1), where the etching time, deposition time, drying temperature, and WCA are denoted as *A*, *B*, *C*, and *R*, respectively.

(1)   R=159.49+4.76×A+1.63×B+1.01×C

The regression equation was analyzed through the variance and coefficient significance tests. The variance analysis results of the response model are shown in Table 3. In RSM analysis, the F value and *p* value Prob > F are always used to evaluate the reliability of the response model and its coefficients. The bigger the model significance F value is and smaller the *p* value Prob > F is, the more significant the impact of factors on WCA. Especially when a *p* value Prob > F is less than 0.01, the influence factor has an extremely significant effect on WCA. From Table 3, the model’s significance level *p*-value Prob > F = 0.0072 < 0.01, indicating that the regression equation is significant at the level of 0.01, which means the regression of this model is quite reliable. Furthermore, the Lack of Fit of the model means whether the model is not applicable. In Table 3, the model’s Lack of Fit is not significant given by the DESIGN EXPERT V8, indicating that the model fitted well with experimental results. It can be seen in Table 4 that the variance expansion factor (VIF) is much smaller than the general requirement of 10, which implies that the collinearity between the regression coefficients is small. The model’s coefficient of variation (C.V.) is 2.10%, which is less than 10%, and the Adeq Precision is 7.7871, which is more than 4, verifying the accuracy of the model. The model shows a linear response relationship that with the increase of each influencing factor, the response value will also increase. Additionally, it can be concluded that etching time has a more significant impact on the WCA.

Considering that the increase in the etching time can lead to further weight loss of the Al fins, the quality loss data of the Al fins is supplemented as a limitation. When predicting the optimal process, to improve the operation accuracy, the etching time is set at 10.5 min, and the maximum value of WCA is chosen as the response value. The most optimal values predicted by the Design-Expert V8 optimization module are the etching time of around 10.5 min, the deposition time of around 48 h, and the drying temperature of around 73.0 °C.

#### 3.1.3. Fabrication of Superhydrophobic Finned-Tube Heat Exchanger

**Figure 3:**
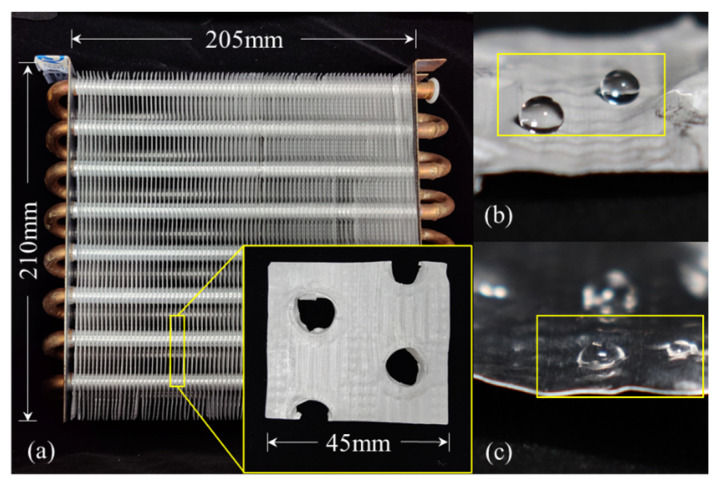
(**a**) Superhydrophobic finned-tube heat exchanger; (**b**) droplets on Al-based superhydrophobic fin; (**c**) droplets on bare Al fin.

The surface modification of the full-size finned-tube heat exchanger was carried out using the optimized process. Figure 3a shows a superhydrophobic finned-tube heat exchanger. Dropped water droplets on the surface of the fins, as Figure 3b,c shows, the preparation method achieved success in full-size heat exchangers, and the hydrophobicity is significantly increased compared with ordinary bare Al fins.

### 3.2. Surface Wettability

**Figure 4:**
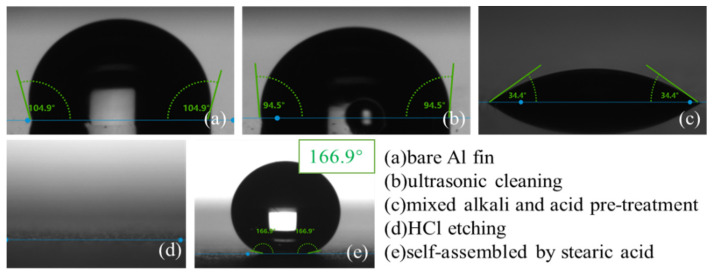
WCAs during the preparation process: (**a**) bare Al fin; (**b**) ultrasonic cleaning; (**c**) mixed alkali and acid pre-treatment; (**d**) HCl etching; (**e**) self-assembled by stearic acid.

**Figure 5:**
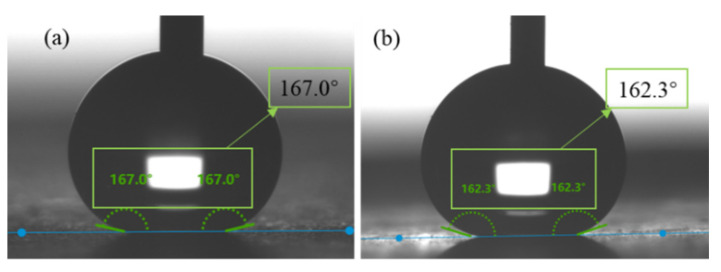
(**a**) Advancing contact angle; (**b**) receding contact angle.

The static WCAs of the Al fins in each process during the fabrication were measured and are shown in Figure 4. Finally, the Al fin showed a static WCA of 166.9°. Figure 5 shows the snapshot of the advancing contact angle and receding contact angle, indicating a CAH of 3.7°, which meets the standards of super-hydrophobicity.

Cassie and Baxter [47] proposed a composite wetting state on the surface composed of air and a solid component due to the rough structure, described as Equation (2):

(2) $$\cos\theta_{c}=f_{s}\cos\theta_{s}+f_{a}\cos\theta_{a}$$

where $\theta_{c}$, $\theta_{s}$, $\theta_{a}$ are the WCAs on the rough surface, smooth solid surface and the air, respectively. *F_s_* and *f_a_* are the area fractions occupied by the solid and the air, respectively. Because of $f_{s}+f_{a}=1$, $\theta_{a}$ = 180°, Equation (2) can be simplified as follows:

(3) $$\cos\theta_{c}=f_{s}\cos\theta_{s}+f_{s}-1$$

According to Equation (3) and $\theta_{c}$ (contact angle of modified Al surface), $\theta_{s}$ (contact angle of bare Al surface), $f_{s}$ is calculated to be 0.065 and $f_{a}$ to be 0.935. This means that air takes up 93.5% of the contact areas in the modified rough structure, which can be considered as the droplet suspended on the modified Al surface. So, when moving on the surface, the potential barrier that the droplet needs to overcome is smaller. This is manifested by a higher WCA and a smaller CAH, which effectively improves the hydrophobicity of the surface.

Then, the surface free energy was obtained by the contact angle method. Measure the contact angle of water and diiodomethane on the modified Al surface, respectively, at six different spots, then calculate the surface free energy using Owens, Wendt, Rabel and Kaelble (OWRK) model [48] and take the average value as a result. The calculation result of the superhydrophobic Al surface is 2.53 mN/m, which is much smaller than that of a bare Al surface (26.33 mN/m).

### 3.3. Surface Morphology and Composition

**Figure 6:**
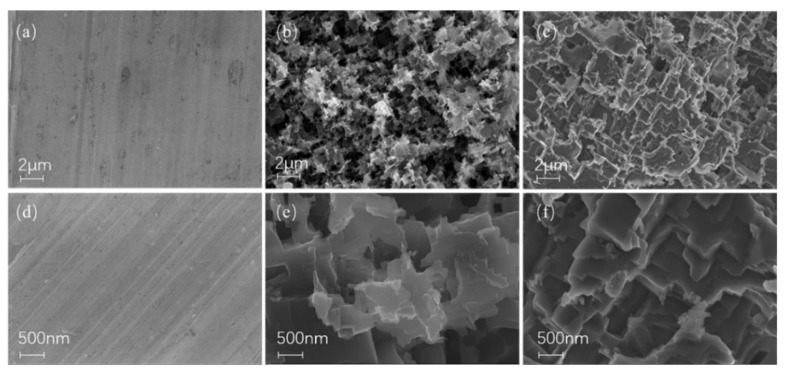
FE-SEM images during the fabrication process. 5000× magnification: (**a**) bare Al fin; (**b**) Al fin etched by HCl solution; (**c**) Al fin modified by stearic acid. 20,000× magnification: (**d**) bare Al fin; (**e**) Al fin etched by HCl solution; (**f**) fin modified by stearic acid.

**Figure 7:**
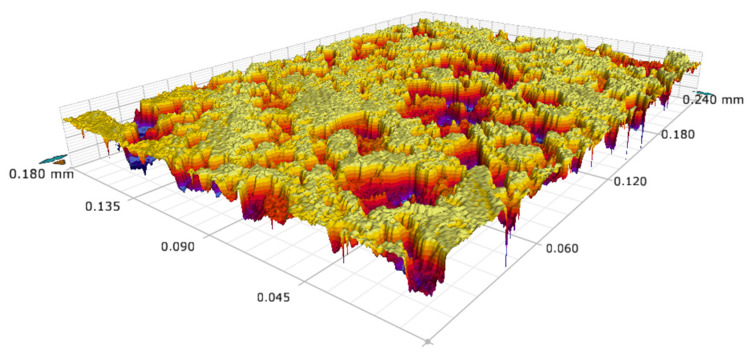
The surface 3D topography image.

The surface morphology of the modified surface was investigated by FE-SEM at 10.00 kV with InLens signal. The FE-SEM images are shown in Figure 6. Figure 6a–c show the surface micro-topography of a bare Al fin, an Al fin etched by HCl solution, and an Al fin modified by stearic acid with 5000× magnification. It can be seen that after etching with an aqueous HCl solution, the relatively smooth surface of the original Al fin appeared with micron-level rough structures. Some small porous structures joined together to form irregular large dimples or grooves. Figure 6d–f display the corresponding images with 20,000× magnification. It can be observed that the micron-sized rectangular-shaped structures were constructed with diameters ranging from 0.5 to 1 μm. Comparing Figure 4b,e and Figure 4a,d, it is clear that micro-sized and nano-sized rough structures were constructed. These cavities with different sizes constituted a hierarchical structure on the Al surface, which is a prerequisite for the water repellency of the surface [49,50]. After the construction of the micro-nano structure, the low surface energy material was attached over the surface, as shown in Figure 4c,f, which made the surface superhydrophobic. The 3D topography of the surface also shows the micro-nano structure, as shown in Figure 7. The mean roughness (Ra) of the modified Al fin was 467.58 nm, and the root-mean-square roughness (Rq) was 595.14 nm. Meanwhile, the maximum peak-to-valley vertical distance (Rt) was 4.095 μm. With the self-assembly of a low surface energy substance, when a water droplet stays on the surface, this structure can provide space to keep the air in the hierarchical roughness. The SEM experiments agree with the calculated results obtained according to the Cassie-Baxter model.

**Figure 8:**
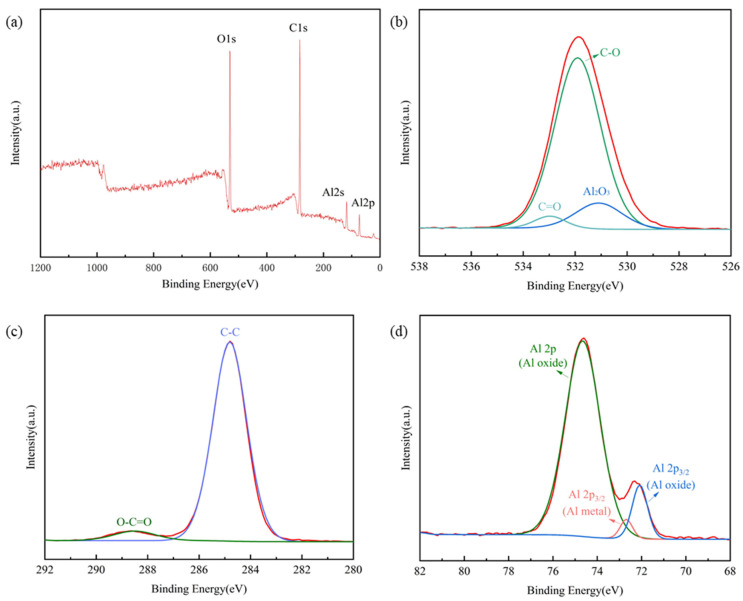
XPS spectrum of superhydrophobic Al alloy surface: (**a**) wide scan survey; (**b**) O 1s spectrum; (**c**) C 1s spectrum; (**d**) Al 2p spectrum.

XPS and FTIR measurements were used to investigate the chemical composition and the chemical bond state of the superhydrophobic Al fins. The XPS spectra were regulated with the binding energy of adventitious carbon (C 1s: 284.8 eV). Figure 8 shows the XPS spectra wide scan survey (a), XPS spectra of O 1s (b), XPS spectra of C 1s (c) and XPS spectra of Al 2p(d), respectively. According to Figure 8a, the sample contained three essential elements, C, O and Al, which means the C and O elements from the stearic acid may have deposited on the Al surface. Then, performed peak fitting to the high-resolution spectra. In Figure 8b, three peaks at a binding energy of 531.9 eV, 533 eV and 531.1 eV represent the C-O bond, C=O bond and Al-O, respectively. In Figure 8c, the peak at 288.5 eV is attributed to ester (O-C=O) from the stearic acid. The peak at 284.8 eV is attributed to the carbon atom in alkyl groups. Figure 8d indicates that the Al 2p has three distinct peaks at 74.6, 72.1 and 72.6 eV. The first two are due to the bonding of Al oxide, and the last one is due to the Al metal.

**Figure 9:**
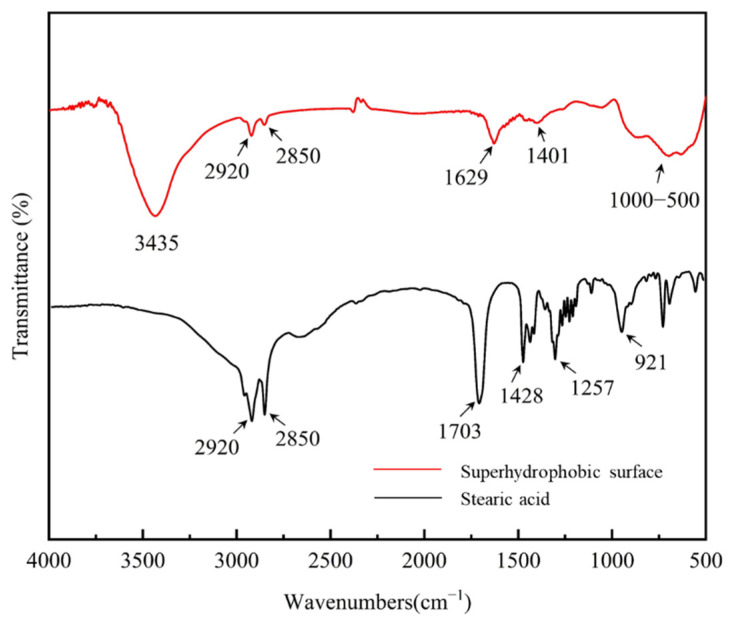
FTIR spectra of the superhydrophobic Al sample and stearic acid.

Since the stearic acid self-assembly layer is relatively thin, the FTIR investigation was performed by scraping the surface substance and then recording the spectra using KBr pellet. Figure 9 shows the FTIR spectra of the superhydrophobic Al sample and stearic acid. The two spectra lines both showed two peaks at 2920 and 2850 cm^−1^, representing the methyl stretching vibration peaks [11,27,51]. The two peaks indicate that the −CH2− groups in stearic acid have been assembled on the superhydrophobic surfaces. The wide peak around 3435 cm^−1^ is assigned to the absorption of Al–OH stretching vibration [28]. In stearic acid spectra, the peaks at 1703 cm^−1^ and 921 cm^−1^ could be considered dimer carboxylic acid and saturated carboxylic acid, respectively. Additionally, the carboxylic acid from stearic acid has absorption peaks around 1428 cm^−1^ and 1257 cm^−1^, which are the result of the coupling of the O-H in-plane bending vibration and the dimer C-O stretching vibration. While in the spectra of superhydrophobic sample, the absorption peaks of carboxylic acid disappear. Instead, the absorption peaks of the carboxylate at 1629 cm^−1^ and 1401 cm^−1^ appear, indicating some kind of reaction occurred with the Al alloy surface and the stearic acid.

According to the principle of chemical reaction, when the Al fin is treated with acid etching, the following reactions (Equation (4)) between the Al surface and HCl solution would be:

2Al (s) + 6HCl (l) → 2AlCl_3_ (s) + 3H_2_(g) (4)

According to the test results of XPS and FTIR, the reason why the Al-O bond and the absorption peak of Al-OH appear is speculated as follows (Equations (5)–(7)):

AlCl_3_ (s) → Al^3+^ (aq) + 3Cl^−^ (aq) (5)

H_2_O (l) → H^+^ (aq) + OH^−^ (aq) (6)

Al^3+^ (aq) + 3OH^−^ (aq) → Al(OH)_3_ (s) (7)

Subsequently, the reactions (Equations (8) and (9)) occur during the self-assembly of stearic acid, so the absorption peak of carboxylate can be observed in the FTIR spectra. Additionally, it indicates that the stearic acid was assembled on the Al-based surface.

CH_3_(CH_2_)_16_COOH (s) → CH_3_(CH_2_)_16_COO^−^ (aq) + H^+^ (aq) (8)

Al^3+^ (aq) + 3CH3(CH_2_)_14_COO^−^ (aq) → Al(CH_3_(CH_2_)_14_COO)_3_ (s) (9)

In all, due to the rough structure and the super-hydrophobicity of the stearic acid alkyl chains, the Al alloy fin is modified with superhydrophobic properties.

### 3.4. Durability Property

#### 3.4.1. Air Exposure Test

**Figure 10:**
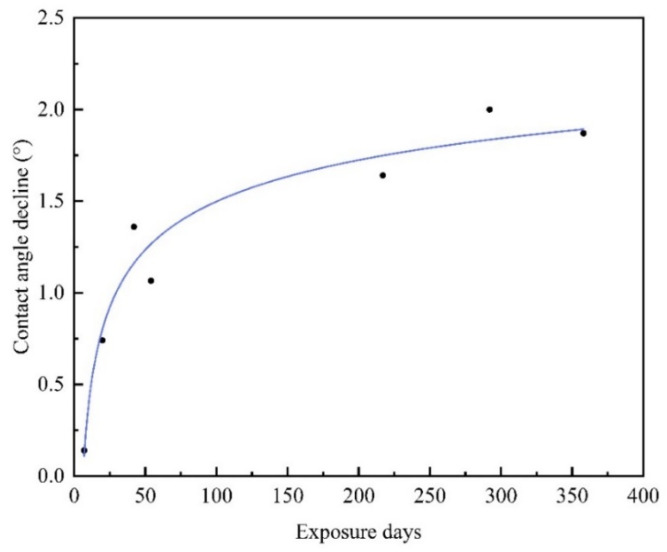
The contact angles decline with air exposure time.

In practical applications, many heat exchangers are exposed to the outdoor environment. The modified surface should remain superhydrophobic in the air for a long time. In this paper, a modified Al-based fin with an initial WCA of 166.6° was placed in the air under room temperature for one year, and the static WCA was measured at regular intervals. The decrease in the WCA with the air exposure time is shown in Figure 10. The contact angle decline was fitted to a logarithmic curve with the exposure time. Defining the exposure day as an independent variable and contact angle decline as the dependent variable, the fitting model is as follows (Equations (10) and (11)):

*y* = 1.613ln [0.550ln(*x*)] (10)

*R^2^* = 1 (11)

If the service life of ASHP is ten years (3650 days), the contact angle decline will be around 2.4°. The contact angle of the surface will be approximately 164.2°, which still represents excellent super-hydrophobicity.

#### 3.4.2. Corrosion Resistance

**Figure 11:**
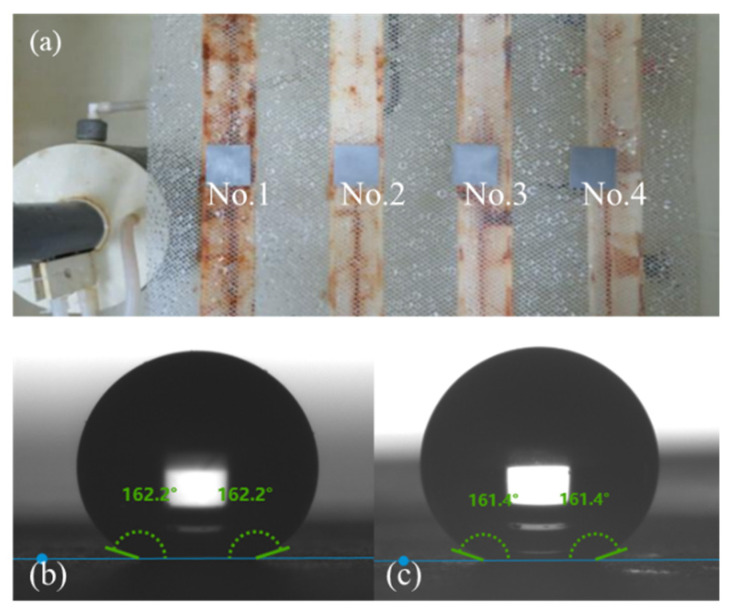
(**a**) Samples in the NSS test box; (**b**) WCA of sample No.4 before corrosion; (**c**) WCA of sample No.4 after corrosion.

Corrosion inevitably occurs on the heat exchangers installed outdoors. Therefore, the accelerated corrosion test of the superhydrophobic surfaces was conducted by NSS. Samples No.1 to No.4 represented the corrosion time12 h, 24 h, 48 h and 72 h, respectively. Figure 11a shows the corrosion state of the four samples in the NSS test box. Figure 11b,c are the WCAs of sample No.4 before and after the NSS test. After 72 h of uninterrupted salt spray deposition, the superhydrophobic surfaces did not show obvious changes. The WCA after corrosion was still maintained above 160°. The modified Al surface showed excellent corrosion resistance in the accelerated corrosion test. In coastal areas and other places with high salt content in the air, it may slow down the corrosion of the heat exchanger surface.

**Figure 12:**
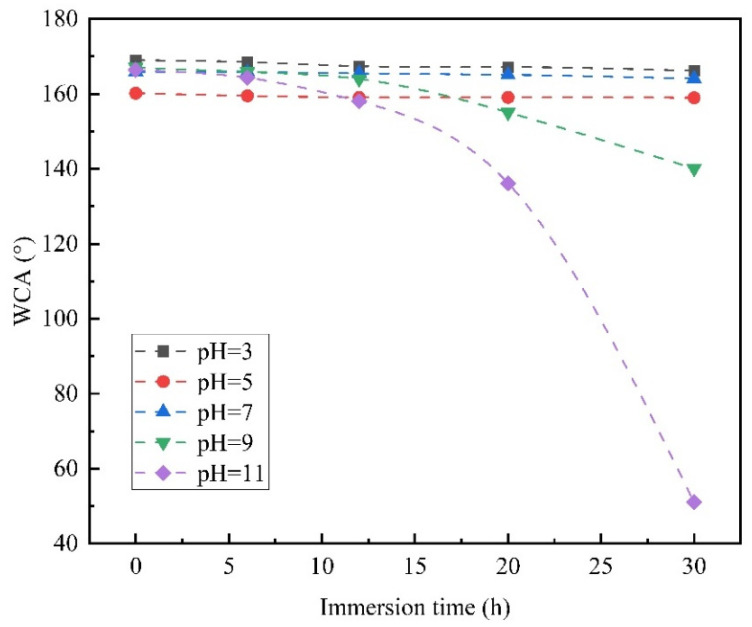
The change of contact angles of samples in solutions of different pH values.

Five Al fins were used for the pH stability test. The parameters of each sample and the pH values of different immersion solutions are shown in Table 5. The contact angles were measured after immersing the five samples for 6, 12, 20, and 30 h. The variation in the contact angle with immersion time in different pH solutions is shown in Figure 12.

The results show that the superhydrophobic Al fins can maintain good hydrophobic properties in neutral to acidic solutions but shows more corrosion in alkaline aqueous solutions and a significant contact angle decline. According to the properties of stearic acid, it is difficult to dissolve in water, so the dissolution of the coating in pure water is very limited, and the contact angle is slightly reduced. Stearic acid cannot be wholly dissociated into ions in water. The acidity coefficient is about 5.75 and can be defined as a kind of weak acid. It is inferred that the stearic acid coating is more stable in a weak acid environment. In the alkaline aqueous solution, the stearic acid coating reacts slowly with alkaline substances, causing the coating to corrode in the aqueous solution with pHs of 9 and 11. Considering that the water vapor in the air can dissolve a small amount of carbon dioxide after condensation, the condensate is neutral to a weakly acidic environment. So, the superhydrophobic Al fins may have good pH stability performance in the normal air environment.

#### 3.4.3. Mechanical Robustness Test

**Figure 13:**
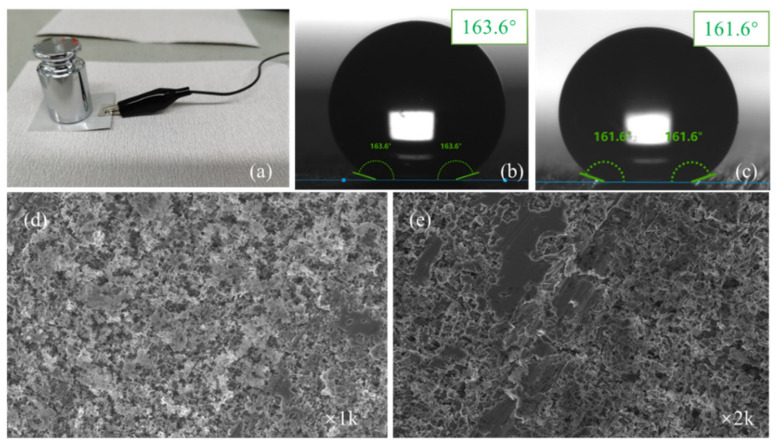
Tangential abrasion test of sandpaper. (**a**) device; (**b**,**c**) WCA before and after wear test; (**d**,**e**) SEM photos of wore surface with magnification times of 1000 and 2000.

Literature has shown that most superhydrophobic/hydrophobic surfaces have poor mechanical durability and are easily affected by wear and scratches. Therefore, the mechanical robustness of superhydrophobic surfaces is another vital evaluation index in practical applications. In this paper, the mechanical robustness of the stearic acid self-assembled surface was initially tested by the tangential abrasion test of sandpaper. The test device is shown in Figure 13a. Figure 13b,c are the static water contact angles before and after wear, respectively. Figure 13d,e are the SEM images of the surface topography after wear with different magnification times. It can be seen that the microstructure of the worn Al fin surface was slightly damaged, and the contact angle test showed that the average WCA decreased slightly. However, the WCA at the worn part still meets the contact angle judgment condition of super-hydrophobicity.

Generally, the superhydrophobic fins have satisfactory durability in harsh operation environments. The WCAs of the fins would be consistent within a superhydrophobic range, even larger than 160° for a long time, due to the stable rough structure and efficient bonding of hydrophobic alkyl chains.

### 3.5. Evaluation of Surface Characteristics

**Figure 14:**
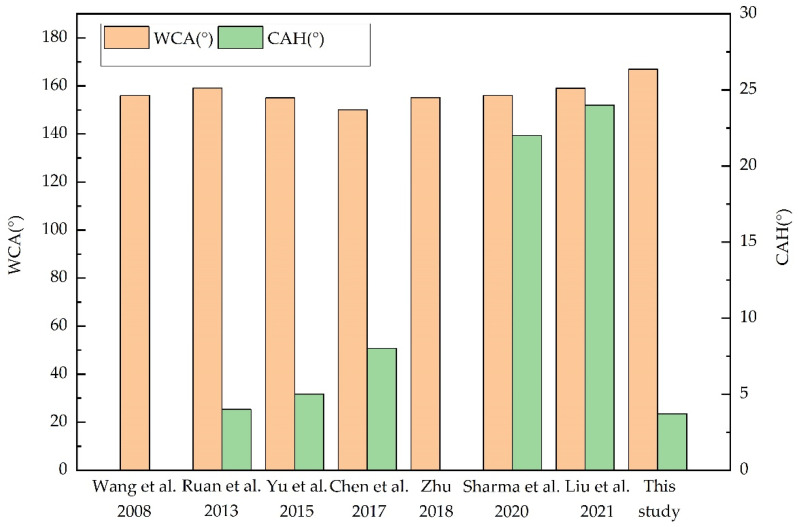
Comparison of wettability results between literature and the present study.

Comparing the fabricated Al fins with the existing studies, a prominent innovation is the improvement of surface wettability, that is, the improvement of the contact angle and the reduction in the sliding angle. Figure 14 shows the comparison of wettability results of different Al-based superhydrophobic surfaces. The unshown columns of CAH are due to the literature data missing. The method reported in this study enables the superhydrophobic Al surface WCA to reach above 160° stably. In addition, the upper limits of the preparation methods in literatures were not discussed. While in this study, the basis of the fabrication parameters was clearly demonstrated, and the process was optimized. In terms of surface corrosion resistance, most studies explained the anti-corrosion mechanism of superhydrophobic surfaces through electrochemical experiments but did not give the maintaining time of superhydrophobic properties. According to the application conditions and characteristics of superhydrophobic heat exchangers, this research provided a durability evaluation combined with engineering applications.

Since the fabrication in this study is conducted in the laboratory, the limitation is whether the existing production mode can be cost-effectively modified to enable large-scale production of superhydrophobic heat exchangers. In addition, how the superhydrophobic heat exchanger can bring energy efficiency improvement to the overall HVAC&R system due to its anti-frost and anti-fouling properties needs to be further studied.

## 4. Conclusions

In this paper, a facile and cost-effective method containing acid etching and stearic acid self-assembly was proposed and improved to fabricate superhydrophobic Al-based finned-tube heat exchangers successfully. The surface characteristics were analyzed, and the surface practicability in the HVAC&R system was verified. The following conclusions can be drawn:

1. The fabrication process and parameters were improved. The single factor experiments of etching solution concentration provided a reasonable HCl solution concentration of 2 mol/L. The RSM and variance analysis determined the response relationship between etching time, deposition time, drying temperature and contact angle. The optimal manufacturing parameters were the etching time in HCl solution of 10.5 min, the self-assembly time in the stearic acid ethanol solution of 48 h, and drying under 73.0 °C.
2. The resulting Al fins have excellent superhydrophobicity. The WCA was 166.9° and the CAH was 3.7°. The area fraction of the air–liquid interface in the superhydrophobic Al surface was 93.5% calculated by Cassie–Baxter model. The free energy of the superhydrophobic fin was 2.53 mN/m.
3. The FE-SEM images and 3D topography analysis showed suitable micro-nano structures, and the XPS and FTIR spectra showed the self-assembly of an alkyl chain, which are essential for the realization of the superhydrophobic surface.
4. A WCA decay model was established for surfaces exposed to air. At a preset service period of 10 years, the WCA dropped would be 2.4°. In evaluating anti-corrosion performance, the modified fins showed good anti-corrosion properties in the NSS accelerated corrosion test, and the surface maintained superhydrophobicity in acidic to neutral environments. In addition, the Al surface still met the evaluation criteria of superhydrophobicity after the abrasion test.

The optimized method may provide theoretical and technical support for the fabrication and application of superhydrophobic full-scale heat exchangers. Since the heat exchange performance of the heat exchangers is vital for the energy-saving and carbon reduction of the HVAC&R system, further research may focus on the investigation and evaluation of heat exchange enhancement of the proposed superhydrophobic heat exchanger.

## Figures and Tables

**Table 1:** Response surface experimental factors and the design values.

Influence Factors	Factor Levels		
	−1	0	1
Etching time/min	10.0	10.5	11.0
Deposition time/h	24	36	48
Drying temperature/°C	70.0	80.0	90.0

**Table 2:** Experimental plan and results.

Run	A. Etching Time/min	B. Deposition Time/h	C. Drying Temperature/°C	Response WCA/°
1	11.0	24	80.0	157.1
2	11.0	48	80.0	168.6
3	10.5	24	90.0	157.1
4	11.0	36	90.0	168.7
5	10.5	24	70.0	161.5
6	10.5	48	90.0	162.6
7	10.0	36	70.0	152.9
8	11.0	36	70.0	161.8
9	10.0	48	80.0	154.3
10	10.5	36	80.0	160.7
11	10.0	36	90.0	155.0
12	10.0	24	80.0	155.9
13	10.5	48	70.0	159.1
14	10.5	36	80.0	155.3
15	10.5	36	80.0	156.2
16	10.5	36	80.0	160.2
17	10.5	36	80.0	164.4

**Table 3:** Variance analysis of response model.

Source	Sum of Squares	Df	Mean Square	F Value	*p* Value Prob > F	
Model	210.78	3	70.26	6.28	0.0072	Significant
A	181.45	1	181.45	16.21	0.0014	
B	21.12	1	21.12	1.89	0.1928	
C	8.20	1	8.20	0.73	0.4075	
Residual	145.53	13	11.19			
Lack of Fit	91.16	9	10.13	0.75	0.6733	Not significant
Pure Error	54.37	4	13.59			
Cor Total	356.31	16				

**Table 4:** Significance of regression coefficient.

Factor	Coefficient Estimate	Df	Standard Error	95% CI		VIF
				Low	High	
Intercept	159.49	1	0.81	157.74	161.25	
A	4.76	1	1.18	2.21	7.32	1.00
B	1.63	1	1.18	−0.93	4.18	1.00
C	1.01	1	1.18	−1.54	3.57	1.00

**Table 5:** Sample parameters in pH stability test.

Sample No.	Initial WCA/°	pH Values of the Aqueous Solutions
1	169.0	3
2	160.2	5
3	165.9	7
4	167.1	9
5	166.4	11

## Data Availability

Data presented in this study are available in this article.
